# Breast Cancer and Autism

**Published:** 2013-03-01

**Authors:** Lisa Radcliff

**Affiliations:** From Oregon Health & Science University, Portland, Oregon

## Abstract

**Case Study**

Amy is a 44-year-old woman with severe autism. She lives with her sister Susan, who is her caregiver and guardian. Amy is ambulatory and able to dress and feed herself. She is a healthy individual with no other significant comorbidities. She walks daily and enjoys her sister’s company. Amy’s life expectancy is greater than 10 years. However, she is difficult to care for medically, as she will not allow a physical examination and strikes out when strangers try to touch her. She is nonverbal and unable to participate in decision-making.

**INITIAL DIAGNOSIS**

Amy has a history of breast cancer diagnosed 2 years ago, originally presenting as a stage I lesion (T2N0) that was palpated by her caregiver while bathing. She underwent right simple mastectomy with sentinel lymph node resection. Susan recalls that the mastectomy was a very challenging ordeal, as Amy kept pulling out IV lines, drains, and dressings. Susan felt that Amy withdrew from her after the procedure as she most likely associated Susan with the cause of the pain, making her role as caregiver more difficult.

Pathology confirmed an invasive ductal carcinoma, moderately differentiated, 2.4 cm, estrogen/progesterone receptor negative, HER2/*neu* negative, with negative surgical margins. Two right axillary sentinel lymph nodes were negative for disease. The standard of care for a patient with these tumor features is surgery plus adjuvant chemotherapy (National Comprehensive Cancer Network [NCCN], 2012). According to the Adjuvant Online! database (2012), Amy’s risk for relapse was approximately 40% without adjuvant treatment; her risk for mortality was approximately 29%. After meeting with a medical oncologist, Amy did not receive adjuvant chemotherapy. According to Susan, she was not offered the choice, and the decision was not explained to them. She was simply told that it was not necessary. Aside from pathology, previous records were unavailable for review.

Medical assessment of Amy’s level of autism reveals marked impairments in the use of multiple nonverbal behaviors such as eye-to-eye gaze, facial expression, body posture, and gestures to regulate social interaction. She exhibits a total lack of development of spoken language, with no attempt to compensate through alternative modes of communication such as gesture. During the visit, she occupies herself with repetitive motor mannerisms. Susan believes that Amy struggles with overstimulation from tactile input. Therefore, she is combative with health-care providers and intolerant of invasive devices. Susan has an intimate understanding of Amy’s ability to communicate her needs and wants through nonverbal changes.

**RECURRENCE**

Approximately 2 months ago, Amy began favoring her right arm and appeared to be in pain when participating in various activities. Susan became aware of Amy’s pain issues by noticing that her posture was slightly altered and she was carrying herself differently. Further investigation with a CT scan showed concern for local disease recurrence involving the axillary lymph nodes. No distant metastases were seen. The standard of care for this diagnosis is surgical resection and consideration of radiation therapy, followed by adjuvant chemotherapy (NCCN, 2012). Susan does not want Amy to undergo further surgery and believes radiation would be too difficult to maneuver. The next best option would be a medical approach with chemotherapy as the main modality.

**DIFFICULT DECISIONS**

If treatment is pursued, the advanced practitioner will need to perform regular examinations and prescribe and monitor chemotherapy. The delivery of therapy, requiring frequent blood draws and IV access, will be a challenge for the health-care staff. The APN is apprehensive about the ability to accomplish these tasks safely given Amy’s limited capacity to participate. The APN is also concerned with how treatment will affect Amy’s life. The APN may have her own individual conflict of morals to contend with, given the limited understanding of the patient vs. nontreatment of a potentially curative malignancy.

Chemotherapy is not an easy task for any patient to undertake, especially for a patient with challenges such as Amy has. Although Susan can give legal consent for her sister, Amy is unable to participate in this decision-making. Susan strongly believes that Amy’s quality of life is much more important than the quantity. Withholding treatment may shorten the natural course of Amy’s life, yet administering chemotherapy will alter the quality of life that she now enjoys without her understanding or consent. Should Amy receive chemotherapy or should Susan refuse treatment on her behalf?

## Medical Indications

Amy has a confirmed breast malignancy that, left untreated, will eventually progress and may shorten her natural lifespan. Physically, she is a good candidate for treatment, lacking any significant comorbidities or physical disabilities that would interfere with the action of chemotherapy. Systemic treatment of locally recurrent breast cancer potentially prolongs survival and palliates disease-related symptoms, but it is not curative. Instead, a sustained remission is the most reasonable outcome. Therefore, interventions with minimal toxicity are preferred in the palliative setting (NCCN, 2012).

The standard chemotherapy regimen for this type of malignancy includes an anthracycline and a taxane-based drug (NCCN, 2012). The potential harmful side effects of these agents include, but are not limited to, infection (2%–55%), bleeding (1%–17%), possible death from immunosuppression (< 1%), congestive heart failure from cardiotoxicity (1%), peripheral neuropathy from neurotoxicity (temporary < 80%, permanent 10%), alopecia (temporary 100%, permanent < 1%), myalgias/arthralgias, fatigue, nausea/vomiting, diarrhea/constipation, taste/smell alteration, anorexia/weight loss, and general malaise (Micromedex, 2012a, 2012b, 2012c). The health-care provider and the guardian must consider these potential negative outcomes when deciding whether or not to choose to pursue treatment.

## Quality of Life

Amy enjoys a high degree of independence. She goes for regular walks and remains quite active. She enjoys spending time outside in a quiet corner of the backyard. Her demeanor changes at the sight of her sister, indicating that she derives pleasure from her sister’s company. She is able to feed herself and Susan remarks that, while a picky eater she really enjoys certain foods. The side effects from chemotherapy will temporarily, and possibly permanently, alter her ability to continue her daily activities.

## Patient Preferences

Amy’s preferences cannot be verbally articulated. As she has been profoundly disabled since birth, no prior preferences regarding medical issues can be ascertained. Her nonverbal communication indicates a desire to be left alone. She clearly does not want examinations or invasive procedures. However, she permanently lacks the mental capacity to grasp the magnitude of this decision. Susan has expressed genuine concern regarding further invasive procedures, based on her postoperative experience after the previous mastectomy. If Amy develops an escalating lack of trust in her caregiver, it would make Susan’s role and ability to care for her sister increasingly difficult and emotionally devastating (Jonsen, Siegler, & Winslade, 2010).

As her legal guardian, Susan is able to consent to therapy. Susan has articulated her desire to maintain Amy’s quality of life, even if that means foregoing quantity. Susan has been informed of the benefits and risks of therapy.

## Contextual Features

Significant consideration must be given to the decision-making burden of the caregiver. Susan has already expressed concerns about her ability to care for her sister if she is further associated with painful or threatening situations. She is struggling with a life-altering, and potentially life-ending, decision for her sister. She is experiencing heightened guilt in choosing to withhold treatment. However, she is incredibly distressed at the idea of restraining her sister and forcibly giving treatment. She has no faith-based objections to medical interventions. Susan’s support system includes her husband and teenage children, who are well accustomed to Amy. However, Susan remains the primary caregiver. She has no other siblings, and her parents are both deceased. Amy will continue to reside with Susan’s family as long as possible.

The nursing staff in the outpatient chemotherapy infusion room has voiced concern in caring for Amy. Several members of the team refuse to restrain Amy in order to deliver care. Their concerns include being hit by the patient, the potential for an accidental needle stick, the potential for a chemotherapy spill, the potential for IV extravasation of a vesicant agent, the disruption of other patients’ treatment, the increased care burden, and the emotional distress of restraining the patient against her will.

The patient advocate from the ethics department reviewed the case. She determined that Amy has an appropriate legal guardian, and therefore it was not necessary for the advocate office to be involved in the decision-making on behalf of the patient. Thus, a formal ethics committee review was not obtained.

## Case Analysis

A "best interest standard" is acceptable in this case (B. Glidewell, personal communication, July 30, 2010). Cantor (2005) defines the best interest standard as the decision or choice that is in the best interest of the patient, independent from the needs or views of the surrogate decision maker, being careful not to undervalue the simple benefits that the disabled person derives from existence. The focus is on the well-being of the patient and consistent with human dignity. This is not to be confused with a substituted judgment standard, as we have no way of knowing Amy’s preference (Hui, 2008). Cantor (2005) recommends considering the degree of physical and mental suffering, the chances of recovery, the nature of the patient’s interaction with her environment, the potential for regaining function, and the level of indignity associated with treatment. Coggon (2008) describes the best interest standard in the context of the Mental Capacity Act, stating "when making decisions for those without capacity, the course of action least restrictive of a person’s rights and freedoms is always to be preferred." From the President’s Commission for the Study of Ethical Problems in Medicine (1983), the best interest standard should include the relief of suffering, the preservation of functioning, and the protection of quality of life.

Martyn (1994) describes a way of evaluating a meaningful life using caregiver interpretation. She argues that gestures, tones, and movements that may seem meaningless to the outside observer may be pertinent to discovery of how that individual experiences life. Susan has confirmed this notion of interpretation through their special relationship by recognizing Amy’s pain symptoms early in the disease process. Susan believes that Amy genuinely enjoys her current lifestyle. Amy’s quality of life would be temporarily and possibly permanently altered by chemotherapy. The level of fatigue associated with chemotherapy would interfere with walking and daily activities. Nausea and taste alteration would dissuade her from the foods that she enjoys. Peripheral neuropathy would change her perception of her environment and can be a permanent disability. Her level of functioning would be altered and her chance of a successful disease outcome is only approximately 50% (NCCN, 2012).

The act of participating in therapy may in itself be severely traumatizing. In order to successfully receive an infusion, she would have to be restrained either physically or chemically. This submission to receive invasive treatment may be considered cruel and unusual, violating her privacy of being. Forcing treatment that exacerbates her fears may actually constitute battery (B. Glidewell, personal communication, July 30, 2010). Restraint without consent or understanding violates Amy’s dignity and thus her protected liberty.

These factors have an impact on the caregiver as well. Susan is afraid of being associated with both the traumatic experience of therapy delivery and the symptoms of treatment. Should the burden on the caregiver be a consideration in this case? Legally, there is no precedent for putting the caregiver’s needs ahead of the patient’s. Philosopher John Hardwig (1997) is an advocate of considering the caregiver burden in life-ending decisions. His argument requires a fairness calculation that assesses the degree of burden and sacrifice required by the third party. Not only does Susan face sacrifices of time and money, she has voiced significant mental anguish regarding treatment of her sister. In this case, the caregiver is the consenting surrogate, so her values are intrinsically woven into the decision-making process.

## Legal Implications

This is a case of giving life-altering treatment in a profoundly mentally disabled person unable to participate in medical decision-making. The United States Declaration of the Rights of the Disabled (section 3) states, "Disabled persons have the right to respect for their human dignity…which implies first and foremost the right to enjoy a decent life as normal and full as possible." From the 14th Amendment to the United States Constitution, all persons are afforded equal protection under the law to include personal liberty. This extends to the right of any person to refuse medical intervention, which translates to an interest in self-determination, well-being, and bodily integrity (Cantor, 2005). Preserving bodily integrity equates to personal dignity. This concept of dignity highlights the role of autonomy in the ethics of medical treatment of the profoundly mentally disabled.

The most prolific legal statute applicable to this situation is derived from case law. The *Superintendent of Belchertown State School & another v. Joseph Saikewicz *(1976) was the case of a profoundly mentally disabled man diagnosed with acute leukemia. The question before the court was whether or not to treat him with life-prolonging chemotherapy. The justices decided to uphold "the right of any person, competent or incompetent, to be spared the deleterious consequences of life-prolonging treatment" (p. 745). With a focus on the patient’s inability to appreciate his situation or verbalize his choice, they argued that

"…principles of equality and respect for all individuals require conclusion that choice exists…a general right in all persons to refuse medical treatment…[therefore] the recognition of that right must extend to the case of an incompetent, as well as a competent, patient because the value of human dignity extends to both (page 745)."

In other words, when considering the basic ethical principles applied to the practice of medicine, profoundly mentally disabled persons have a right to treatment under the auspices of equality and autonomy and the right to refuse treatment as well.

## Case Resolution

After considering the possible outcomes and discussing the issues with delivering therapy, Susan decides that Amy should not receive treatment. She wants Amy to continue to enjoy the life she currently leads with minimal interruption. As her terminal illness progresses, the pain associated with malignancy may begin to interfere with her current level of activity. Avoidance of suffering can be achieved by creating a plan for palliative care and eventual hospice in advance of the natural timeline of the disease (B. Glidewell, personal communication, July 30, 2010). The social worker and palliative care team have been consulted on the case. They are recommending an aggressive pain regimen and assistance in the home when Amy begins to decline. Radiation oncology has also been consulted. If Amy develops a painful bony lesion, as is common with metastatic breast cancer, she could receive a small dose of radiation for palliative pain control with minimal difficulty. The health-care team believes a good outcome is quite possible in this case and concurs with Susan’s decision to withhold treatment.

## Conclusion

When evaluating a patient with a severe developmental disability for his or her fitness for chemotherapy, the oncology advanced practitioner should strongly consider the patient’s quality of life. Utilization of their institution’s advocacy office and/or an ethics committee case review can be helpful in many situtations. Inclusion of the entire care team and the patient’s support system, including the primary caregiver, is paramount for a successful outcome. Always remember that the patient has both the right to be considered for treatment regardless of his or her disability and the right to not receive therapy.
